# Altered NRF2 signalling in systemic redox imbalance: Insights from non-communicable diseases

**DOI:** 10.1016/j.redox.2025.103891

**Published:** 2025-10-10

**Authors:** Monika Jakubowska, Vera Marisa Costa, Wojciech Krzeptowski, Pia Pužar Dominkuš, Marlene Santos, Birsen Can Demirdöğen, Şermin Genç, Ioannis P. Trougakos, Katja M. Kanninen, Brigitte M. Winklhofer-Roob, Ian M. Copple, Antonio Cuadrado, Vita Dolžan, Christina Morgenstern

**Affiliations:** a Malopolska Centre of Biotechnology, Jagiellonian University in Krakow, Krakow, Poland; b Associate Laboratory i4HB—Institute for Health and Bioeconomy, Faculty of Pharmacy, University of Porto, Porto, Portugal; c UCIBIO-Applied Molecular Biosciences Unit, Laboratory of Toxicology, Department of Biological Sciences, Faculty of Pharmacy, University of Porto, Porto, Portugal; d Maj Institute of Pharmacology, Polish Academy of Sciences, Department of Drug Addiction Pharmacology, Laboratory of Neuropharmacology and Epigenetics, Krakow, Poland; e Pharmacogenetics Laboratory, Institute of Biochemistry and Molecular Genetics, Faculty of Medicine, University of Ljubljana, Ljubljana, Slovenia; f REQUIMTE/LAQV, Escola Superior de Saúde, Instituto Politécnico do Porto, Rua Dr. António Bernardino de Almeida, Porto, Portugal; g Molecular Oncology & Viral Pathology, IPO-Porto Research Center (CI-IPOP), Portuguese Institute of Oncology, Porto, Portugal; h Department of Biomedical Engineering, TOBB University of Economics and Technology, Ankara, Türkiye; i İzmir Biomedicine and Genome Center, Izmir, Türkiye; j Department of Cell Biology and Biophysics, Faculty of Biology, National and Kapodistrian University of Athens, Athens, Greece; k A.I. Virtanen Institute for Molecular Sciences, University of Eastern Finland, Kuopio, Finland; l Institute for Molecular Biosciences, University of Graz, Graz, Austria; m Department of Pharmacology & Therapeutics, Institute of Systems, Molecular & Integrative Biology, University of Liverpool, Liverpool, UK; n Department of Biochemistry, School of Medicine, Universidad Autónoma de Madrid, Madrid, Spain; o Instituto de Investigación Sanitaria La Paz (IdiPaz), Instituto de Investigaciones Biomédicas “Sols-Morreale” UAM-CSIC, Madrid, Spain; p Centro de Investigación Biomédica en Red de Enfermedades Neurodegenerativas (CIBERNED), Madrid, Spain; q Vienna Airway Lab, Department of Otorhinolaryngology, Medical University of Vienna, Vienna, Austria

**Keywords:** Biomarker, NRF2, Non-communicable diseases, Redox imbalance, Oxidative stress, Transcription factor

## Abstract

The balanced activity of the cytoprotective transcription factor NRF2 is central for maintaining redox, metabolic-energetics, and proteome homeostasis, as well as for regulating inflammatory responses, among other functions. Activated NRF2 regulates the expression of hundreds of genes containing antioxidant response elements (AREs) or electrophile response elements (EpRE) in their regulatory regions, often promoting cytoprotection under stress conditions and contributing to defence against various pathologies and non-communicable diseases (NCDs). The products of increased NRF2 activity, detected systemically, may originate from either the white blood cells, the cells of the vasculature or tissue-derived products that could be secreted into biological fluids. Therefore, assessing basal and inducible NRF2 activity in blood or other biofluids is crucial for inferring NRF2 responses in local and often inaccessible tissues. In previous work, we identified a panel of six biomarkers - Glutamate-cysteine ligase catalytic subunit (GCLC), Glutamate-cysteine ligase modifier subunit (GCLM), Haem oxygenase 1 (HMOX1), NAD(P)H quinone dehydrogenase 1 (NQO1), Sulfiredoxin 1 (SRXN1), and Thioredoxin reductase 1 (TXNRD1) - as indicators of NRF2 activity. In the current study, we assess their utility in a clinical setting to measure NRF2 activation in a disease context. Here we discuss findings on how NRF2 activity in accessible human samples can reveal its involvement in various NCDs and its connection to clinical aspects such as diagnosis, disease progression and response to therapy.

## Abbreviations

AAA: Abdominal aortic aneurysm
ACTB: Actin beta
AD: Alzheimer disease
ARE: Antioxidant response element
ASD: Autism spectrum disorder
BMI: Body mass index
BPD: Bronchopulmonary dysplasia
Bromocriptine-QR: Bromocriptine quick release
BSH: Broccoli sprout homogenate
CAD: Coronary artery disease
CD: Cluster of differentiation
CKD: Chronic kidney disease
CML: Chronic myeloid leukaemia
c-LDL: Circulating low-density lipoprotein
COPD: Chronic obstructive pulmonary disease
CPB: Cardiopulmonary bypass
CVDs: Cardiovascular diseases
DE: Diesel exhaust
DLBCL: Diffuse large B-cell lymphoma
DOI: Digital object identifier
ELISA: Enzyme-linked immunosorbent assay
EpRE: Electrophile response element
FDRT: First-degree relative with Type 2 diabetes mellitus
GCLC: Glutamate-cysteine ligase catalytic subunit
GCLM: Glutamate-cysteine ligase modifier subunit
GEO: Gene Expression Omnibus
GFR: Glomerular filtration rate
GR: Glutathione reductase
GSH: Glutathione (reduced)
HbE: Haemoglobin E
HMOX1: Haem oxygenase 1
HPA: Human Protein Atlas
HSFA: High-saturated fatty acid
IARC: International Agency for Research on Cancer
KDIGO: Kidney Disease Improving Global Outcomes
KEAP1: Kelch-like ECH-associated protein 1
MeSH: Medical Subject Headings
NAC: *N*-acetylcysteine
NCD: Non-communicable disease
NGS: Next-generation sequencing
NQO1: NAD(P)H quinone dehydrogenase 1
NRF2: Nuclear factor erythroid 2-like factor 2
nTPM: Normalised transcripts per million
PBMC: Peripheral blood mononuclear cell
PD: Parkinson disease
PDT: Periodontitis
PRISMA: Preferred Reporting Items for Systematic reviews and Meta-Analyses
PMID: PubMed identifier
RCT: Randomized, placebo-controlled trial
RPL41: 60S ribosomal protein L41
RS: Resistant starch
RT-qPCR: Quantitative reverse transcription polymerase chain reaction
SCD: Sickle cell disease
SFN: Sulforaphane
SRXN1: Sulfiredoxin 1
T2DM: Type 2 diabetes mellitus
TBP: TATA-box binding protein
tRES-HESP: *Trans*-resveratrol and hesperetin
TXNRD1: Thioredoxin reductase 1
VLBW: Very-low-birth-weight infants

## 1 Introduction

**Fig. 1:**
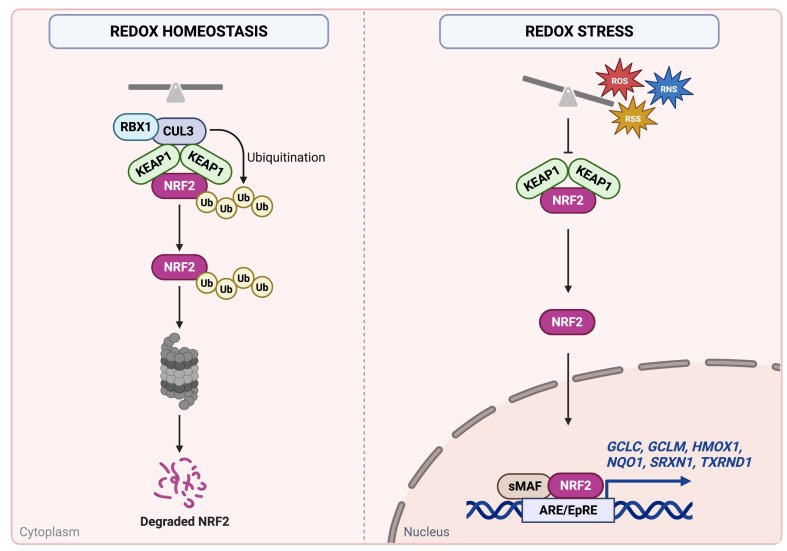
**The NRF2 signalling pathway in redox homeostasis and in redox stress conditions.** Under homeostatic conditions, NRF2 is targeted for proteasomal degradation by the KEAP1/CUL3/RBX1 complex (left scheme). Upon activation by reactive oxygen species (ROS), reactive nitrogen species (RNS) or reactive sulfur species (RSS), NRF2 accumulates in the nucleus and drives the expression of cytoprotective genes, such as *GCLC, GCLM, HMOX1, NQO1, SRXN1* and *TXNRD1* (right scheme).

Non-communicable diseases (NCDs) such as neurological, respiratory tract, cardiovascular disorders, metabolic diseases, chronic kidney diseases, hereditary blood disorders - like β -thalassemia and sickle cell anaemia - and cancer constitute the leading causes of morbidity and mortality worldwide; and their incidence is projected to rise in the coming decades with global ageing population and an increase in environmental stressors being dominant factors [1]. A hallmark of many NCDs is the presence of persistent redox imbalance and chronic inflammation, which can promote disease onset and progression [2]. In this context, the nuclear factor erythroid 2-like factor 2 (NRF2) has gained interest as a potential regulator of cellular defence against oxidative damage [3]. Consequently, aberrant NRF2 signalling is a common feature of several pathological conditions [[4], [5], [6], [7]]. By modulating the expression of numerous cytoprotective genes, NRF2 orchestrates detoxification pathways, redox balance, and metabolic homeostasis, rendering it a pivotal target for both mechanistic studies and even therapeutic interventions in NCDs [8] (Fig. 1).

A recent comprehensive review [9] highlighted six genes: Glutamate-cysteine ligase catalytic subunit (*GCLC*)*,* Glutamate-cysteine ligase modifier subunit (*GCLM*)*,* Haem oxygenase 1 (*HMOX1*)*,* NAD(P)H quinone dehydrogenase 1 (*NQO1*)*,* Sulfiredoxin 1 (*SRXN1*)*,* and Thioredoxin reductase 1 (*TXNRD1*)*,* which can serve as a robust panel of markers that are directly regulated by NRF2 across multiple tissues and cell types. Collectively, these genes encompass key steps in redox homeostasis, including glutathione synthesis (*GCLC, GCLM*), haem catabolism (*HMOX1*), quinone reduction (*NQO1*), sulfiredoxin function (*SRXN1*), and thioredoxin regeneration (*TXNRD1*). Thus, these six markers may act as a minimal gene/protein expression signature indicating redox imbalance and might reflect NRF2 pathway overall activity.

Evidence suggests that NRF2 activation occurs in various tissues during stressful conditions as well as in the context of NCDs, making the clinical assessment of NRF2 activation in peripheral blood [[10], [11], [12]] or other accessible biological samples feasible. Consequently, biofluid-based assays of these NRF2 targets, including cellular components such as peripheral blood mononuclear cells (PBMCs), hold significant promise for clinical application. By tracking shifts in the oxidative defence status, clinicians could identify high-risk patients earlier in the disease trajectory, monitor patients’ response to therapeutic interventions, antioxidant supplementation, or lifestyle modifications, and refine prognostic assessments in a range of NCDs [11]. Moreover, targeted modulation of the NRF2 pathway may offer disease-modifying strategies, as illustrated by experimental studies showing that pharmacological NRF2 activation can mitigate pathological oxidative damage and improve clinical outcomes [13,14]. Validating these biomarkers in clinical settings is essential to fully harness their translational value. Taking this into perspective, the primary aim of this review is to evaluate the evidence of the coordinated regulation of the NRF2 targets (*GCLC, GCLM, HMOX1, NQO1, SRXN1*, and *TXNRD1)* in the early diagnosis, overall prognosis, therapeutic response as well as aetiology of NCDs.

## 2 Methods

### 2.1 Design, search strategy, and eligibility

**Fig. 2:**
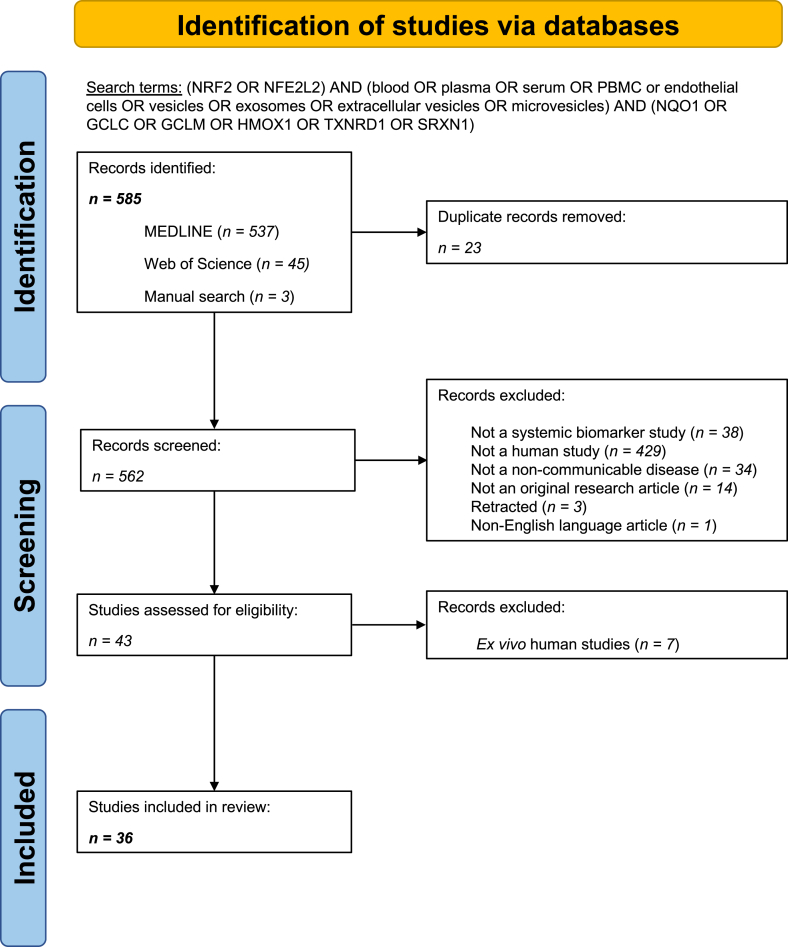
**PRISMA diagramme**. Structured approach of identifying and screening publications to be used in this article from PubMed and Web of Science according to the PRISMA guidelines.

This systematic review was conducted following the Preferred Reporting Items for Systematic Reviews and Meta-Analyses (PRISMA) guidelines [15]. PubMed, including MEDLINE and non-MEDLINE-indexed journals, and the Web of Science (WoS) databases were searched using the jointly defined terms: (NRF2 OR NFE2L2) AND (blood OR plasma OR serum OR PBMC or endothelial cells OR vesicles OR exosomes OR extracellular vesicles OR microvesicles) AND (NQO1 OR GCLC OR GCLM OR HMOX1 OR TXNRD1 OR SRXN1). For the WoS search, the keyword “human” was added to the stated search terms, whereas in PubMed, the filter option “human” was applied. The literature search was conducted between September and October 2024 without any restrictions on publication dates. In addition, three manuscripts of interest were added manually to the paper corpus. Three authors (MJ, PPD, WK) screened the identified records (n = 585). After the initial search all duplicates were removed and additional studies were excluded based on the following criteria: i) studies did not investigate systemic biomarkers, ii) studies were conducted in organisms other than humans, or were *ex vivo* studies of human cells/tissues, iii) studies did not investigate NCDs, iv) were not original research articles (e.g. literature review articles, perspective/opinion articles, and others), v) were retracted, or vi) published in languages other than English. The workflow of our systematic review is shown in Fig. 2. Following study selection, we developed a plan for extracting necessary data and assigned responsibilities.

### 2.2 Data extraction

The selected publications were categorised according to disease type into neurological diseases, respiratory tract diseases, metabolic diseases, cardiovascular diseases, chronic kidney diseases, hereditary blood disorders, cancer, and “other” conditions. Following the literature search, relevant data were extracted from the selected studies, including PubMed and digital object identifiers (PMID and DOI, respectively), authors, year of publication, disease reported using the Medical Subject Heading (MeSH) nomenclature, biological sample type, method(s) of NRF2 biomarker detection, number of cases and controls, and information about the detection of NRF2-regulated genes/proteins: *GCLC, GCLM, HMOX1, NQO1, SRXN1* and *TXNRD1*. Analyses were supported by data on the direction of the marker's expression, along with the statistical significance of the results. The extracted data were cross-checked by a second author to ensure accuracy.

### 2.3 Data visualisation

For visualisation of the results, R [v4.4.1 (2024-06-14, R Foundation for Statistical Computing, Vienna, Austria)] with RStudio (v2024.09.0 + 375) was used, and plotting was performed with the *ggplot2* (v3.5.2) library.

Fig. 4 was created using MS PowerPoint, BioRender (artwork licensed under agreement number: PL28CWK3PN), and GraphPad Prism 10. The single-cell transcriptomic data in this figure were retrieved from the Human Protein Atlas (HPA) database (proteinatlas.org), under the Creative Commons Attribution-Share Alike 4.0 International License. Specifically, the blood cell type expression overview (Fig. 4A) of *GCLC, GCLM, HMOX1, NQO1, SRXN1*, and *TXNRD1* shows the averaged RNA-seq data from human granulocytes (basophils, eosinophils, neutrophils), monocytes (classical, non-classical, intermediate), T cells (T-regs, gdT-cells, MAIT T-cells, naïve, and memory CD4 T-cells, naïve, and memory CD8 T-cells), B cells (naïve, and memory), dendritic cells (plasmacytoid, myeloid), and natural killer cells, accessed from the Tabula Sapiens Consortium molecular cell atlas [16] and the Human Protein Atlas reference dataset [17]. For genes of interest, expression levels above a threshold of ≥1 normalised transcripts per million (nTPM) were averaged for a specific blood cell type and presented as numerical values alongside heat maps depicting the percentages of the highest expression (100 %) of each gene, shown as transparency gradients. Consensus protein expression data (Fig. 4B) show total PBMC levels of: GCLC, GCLM, HMOX1, NQO1, SRXN1, and TXNRD1.

Figure panels were arranged in Affinity Publisher 2 (v2.6.2).

## 3 Results

### 3.1 Systematic literature review and study characteristics

**Fig. 3:**
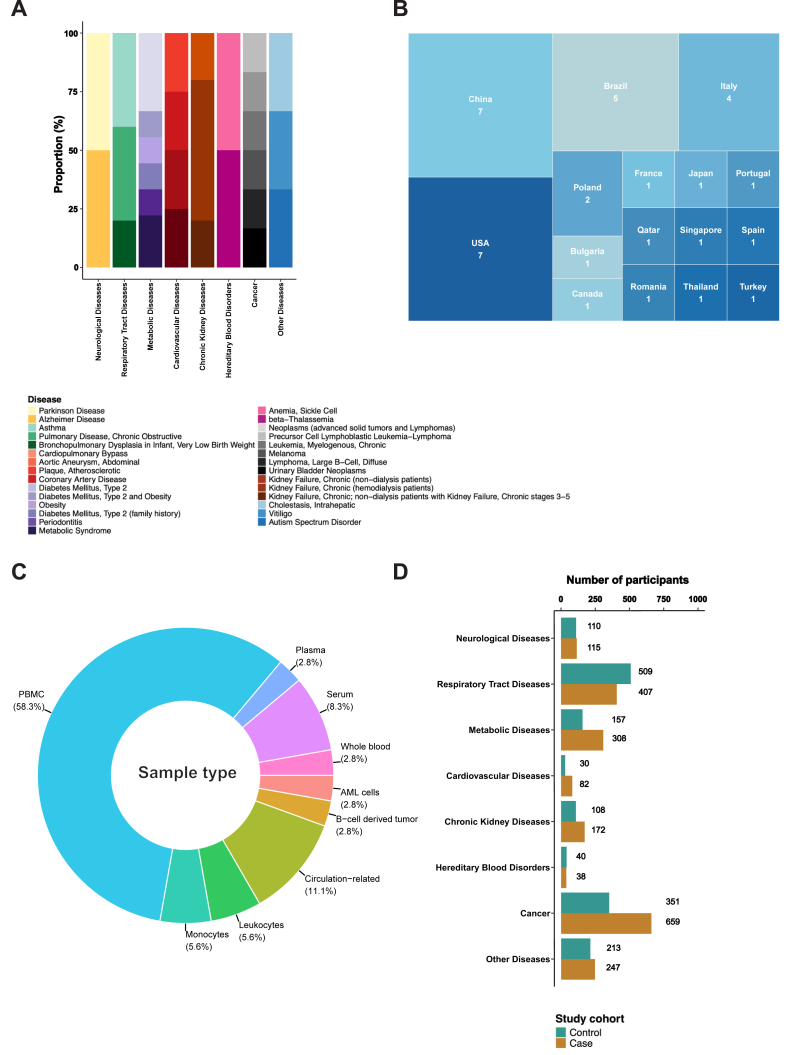
**Summary statistics on the studies included in the systematic review.** (A) Bar chart showing the proportions of specific diseases within each disease category included in the review. (B) Tree map representing the countries conducting and publishing the reported studies. (C) Proportional doughnut plot visualising the different sample types recorded in the review. (D) Bar chart highlighting the number of cases and controls involved in the studies and split per disease category.

We screened 585 studies and selected 36 publications for this review (Fig. 2, Table S1). Since we were particularly interested in identifying NRF2 activity signatures common across different NCDs, we stratified the data into defined disease categories. Most studies were identified as reporting data on metabolic diseases (n = 9), followed by cancer (n = 6), diseases of the respiratory tract (n = 5) or kidney (n = 5), cardiovascular diseases (n = 4), neurological diseases (n = 2), hereditary blood disorders β-thalassemia and sickle cell disease (n = 2), and other diseases (n = 3) (Table S1). Fig. 3A visualises the specific diseases within each category and their proportions.

**Fig. 4:**
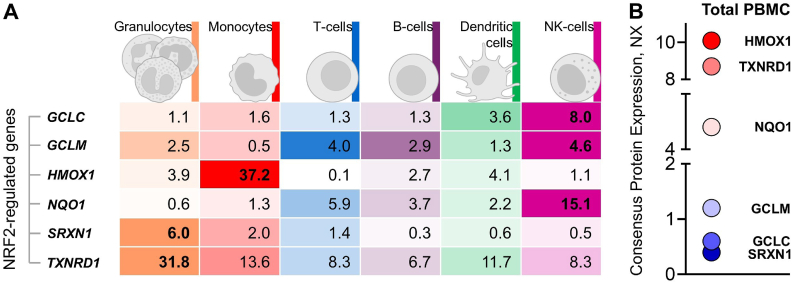
**NRF2-regulated genes in human blood cells.** (A) The single-cell transcriptomic data (RNA-seq) retrieved from the Human Protein Atlas (HPA) database (proteinatlas.org) on NRF2-related biomarkers *GCLC, GCLM, HMOX1, NQO1, SRXN1,* and *TXNRD1* expression in granulocytes (orange), monocytes (red), T cells (blue), B cells (violet), dendritic cells (green), and natural killer cells (magenta), accessed from the Tabula Sapiens Consortium molecular cell atlas [16] and the Human Protein Atlas reference dataset [95]. For each gene, the averaged expression levels, above a threshold of ≥1 normalised transcripts per million (nTPM), are presented as numerical values alongside heat maps depicting the differences in gene expression (full colour for the highest gene expression, and graded transparency for lower expressions) in blood cells. (B) Consensus protein expression data in total peripheral blood mononuclear cells (PBMC) levels of: GCLC, GCLM, HMOX1, NQO1, SRXN1, and TXNRD1.

Geographically, the countries with the highest number of reported studies were USA (n = 7) and China (n = 7), followed by Brazil (n = 5), Italy (n = 4), and Poland (n = 2); another 11 studies reported data from a single country (Fig. 3B).

Regarding the detection methodology, enzyme-linked immunosorbent assay (ELISA) was most often employed to measure NRF2-target proteins, while Western blot was used less frequently to evaluate alterations in protein expression (Table S1). Expression levels of NRF2 target genes, which indicate NRF2 activity, were primarily measured in PBMCs, serum, whole blood, other isolated blood cells (such as monocytes and leukocytes), as well as in plasma by quantitative reverse transcription polymerase chain reaction (RT-qPCR). Additional sample types included buffy coat, other circulation-associated tissues (e.g., aneurysmal tissue and human atherosclerotic plaques), and blood-derived cancer samples, particularly B-cell tumours (Fig. 3C).

The levels of hierarchical evidence (based on [18]), provided by the studies for clinical decision were noted (Table S1). For different types of studies, the levels of evidence were indicated as 1B for a well-designed individual randomized, placebo-controlled trial (RCT) (not a pilot or feasibility study with a small sample size), 2B for an individual prospective cohort study, low-quality RCT or two-group non-randomized study, and 3B for an individual retrospective case-control study, one-group pre-test or post-test study or cohort study, with 1B representing the highest level of evidence of individual studies (Table S1). This literature search retrieved two studies at the 1B level, in metabolic diseases or chronic kidney diseases (Table S1); and four studies at 1B–2B levels: two in diseases of the respiratory tract, and one each of metabolic diseases or chronic kidney diseases (Table S1).

The participants in the analysed studies are shown in Table S1 and were divided into cases and controls. The highest numbers of subjects were available within the cancer section, followed by respiratory tract diseases, and metabolic diseases. In the former category, the cases group outnumbers the control group (Fig. 3D). The type of controls can be inferred from Table S1, which refers to subjects pre- and post-treatment, healthy controls, healthy tissue adjacent to diseased tissue, as well as a lack of control amongst others.

Altered levels of the NRF2 target genes/proteins were frequently found in the presence of diseases and considered a common signature of redox imbalance or chronic inflammation, leading to distorted health. However, it should be noted that a significant association with a disease does not constitute proof of causality. The relationship between redox imbalance and disease pathogenesis can be categorised in three ways: as a primary cause, as a secondary consequence, contributing to disease exacerbation or progression, or as a tertiary epiphenomenon, where oxidative stress is a correlate of the disease state but its reduction does not alter the disease course [19]. In a similar setting, changes (or absence thereof) in response to treatment do not prove efficacy (or absence thereof) if not studied in an appropriately powered RCT. Randomisation is crucial to exclude external influences on a given study group, while in underpowered studies, potential effects may not be detected due to type II error, even though a statistical difference between treatment and control groups may exist.

### 3.2 Neurological diseases

Neurological disorders, including neurodegenerative diseases, are the leading cause of disability and the second leading cause of death worldwide. In 2021, an estimated 3.4 billion individuals (43.1 % of the world population) were affected by conditions that harm the nervous system, damaging the brain and impairing cognition and motor functions, among other effects [20].

The brain is particularly vulnerable to oxidative stress due to its high metabolic rate and oxygen consumption [21] as well as its lipid richness and relatively low amount of antioxidant defences [22]. Several neurological conditions, including the age-related Alzheimer (AD) and Parkinson (PD) diseases are associated with elevated oxidative load and consequently redox imbalance.

Oxidative stress is increasingly regarded as a crucial element in the development of dementia - particularly AD, which affects 32 million people worldwide [23] and is characterized by cognitive decline, memory impairment, and neuronal damage. AD-associated cognitive impairment arises from the abnormal accumulation of proteins in the brain, concomitant with brain cell degeneration and increased levels of markers of oxidative stress, including proteome oxidation, DNA/RNA oxidation, and increased levels of by-products of lipid peroxidation [21]. Molecular alterations occurring in the brain and the nervous system can be monitored in the blood, using newly developed ultra-sensitive methods. Indeed, by utilising a targeted transcriptomic approach, alterations in 12 redox-related genes have been identified in the peripheral blood samples of patients with mild AD [24]. The authors concluded that the increased expression of *HMOX1*, *NQO1*, and *SRXN1* (all p < 0.01), among these redox-related genes, provides evidence of disruption in the NRF2 pathway in the blood of AD patients.

PD is characterized by motor and non-motor symptoms and associated with the degeneration of dopaminergic neurons in the substantia nigra region of the brain and the abnormal accumulation of alpha-synuclein. Global estimates in 2019 indicated that 8.5 million individuals are affected by PD [25]. Although the precise mechanisms underlying PD remain unclear, research suggests that oxidative stress plays a key role, with elevated levels of oxidized proteins, DNA, and lipids, alongside reduced levels of the cellular antioxidant, reduced glutathione (GSH), which are found in PD-affected brains [21]. Systemic activation of the NRF2 pathway has also been demonstrated in PD [26] in a study involving 45 PD patients and 40 control subjects, revealing an increase in the mRNA levels of glutamate-cysteine ligase (GCL), (p < 0.01) as well as elevated protein levels of NQO1 (p < 0.01) detected in leukocytes (number of controls and cases, each of 38). The correlation noted between disease duration and *NRF2* transcript levels suggested that sustained oxidative stress accompanies the presence of the disease.

### 3.3 Respiratory tract diseases

Respiratory tract diseases affect the airways, including the nose, bronchi, and lungs, and range from acute infections to chronic conditions like asthma, chronic obstructive pulmonary disease (COPD), and lung cancer. Key risk factors include tobacco smoke, air pollution, occupational hazards, malnutrition, low birth weight, and early lung infections. The NRF2-KEAP1 signalling pathway plays a pivotal role in various respiratory tract diseases through protecting the upper and lower airways against oxidative damage [27].

Asthma involves reversible airflow obstruction and increased airway sensitivity to allergens or infections, causing symptoms like shortness of breath, coughing, and wheezing [28]. It represents the most prevalent chronic respiratory disease, especially among children, and it affects over 300 million individuals worldwide [29]. The global prevalence of asthma [30] is 9.1 % among children, 11.0 % among adolescents, and 6.6 % among adults [31].

In a randomized crossover, double-blind study, 13 asthmatic patients were exposed to diesel exhaust (DE, 300 μg of PM 2.5/m^3^) or filtered air with and without supplementation of *N*-acetylcysteine (NAC) [32]. MicroRNA profiling of peripheral blood samples revealed an upregulation of miR-144, indicative of systemic oxidative stress upon DE exposure. This increase was attenuated by supplementation with NAC, a well-established precursor of l-cysteine, that elevates intracellular glutathione levels, thereby conferring indirect antioxidant activity. NRF2, along with the enzymes NQO1 and GCLC, were assessed and found to be negatively associated with miR-144 levels (p < 0.05, p < 0.01, p < 0.001, respectively) [32].

Similarly, a double-blind, placebo-controlled, randomized study by Sudini et al. aimed at improving airway inflammation in adults with asthma by supplementation with broccoli sprouts, a rich source of the NRF2 inducer sulforaphane (SFN) [33]. Although an increase of SFN level was observed in serum, the transcript levels of the NRF2 target genes *HMOX1*, *NQO1*, *GCLC,* and *GCLM* were not significantly different between the groups in nasal epithelial cells, and the levels of those genes in circulating PBMCs were also not significantly different between the groups.

On the contrary, in COPD – a complex disease involving airflow obstruction – two studies by Pasini et al. reported an upregulation of NRF2 target genes when comparing patients with healthy controls. In a study of ex-smokers with and without COPD, the expression of *HMOX1* (p < 0.001) was significantly upregulated in PBMCs from individuals suffering from COPD, thus emphasizing the role of the NRF2 pathway as a systemic response aiming to reduce the burden of the disease-induced oxidative stress [34]. Similarly, in subjects with mild to moderate COPD, *HMOX1* and *GCLC* were significantly upregulated in PBMCs (p < 0.05) as compared to the control COPD-free group [35].

Bronchopulmonary dysplasia (BPD) is a condition that affects very-low-birth-weight infants (VLBW, birth weight <1500 g) and leads to lung injury and concomitant oxidative stress. In an association study with ARE variants, Sampath et al. revealed that *NQO1* is markedly upregulated in circulation-related samples (p < 0.01) of infants with severe BPD [36].

Despite the respiratory tract's constant exposure to increased (*versus* human body tissues) oxygen levels, research on the NRF2 pathway's potential to mitigate these effects remains limited and warrants further investigation.

### 3.4 Metabolic diseases

In 2021, an estimated 529 million people worldwide were living with diabetes, and this number is projected to exceed 1 billion by 2050 [37]. Type 2 diabetes mellitus (T2DM) is one of the leading causes of death and disability globally, affecting individuals across all countries and sexes.

T2DM, a metabolic disorder primarily characterized by impaired insulin sensitivity, secretion and action is regarded as a rapidly growing global pandemic. Recent research has significantly enhanced our understanding of the role of inflammation and oxidative stress. Furthermore, the involvement of the adipose tissue, as well as the gut microbiota has gained considerable attention. Underlying T2DM, inflammation, oxidative stress, and impaired insulin production and secretion among insulin resistance have been extensively studied, along with the identification of potential biomarkers that may assist in future treatment and prognostic strategies [38]. These findings also highlight a connection with NRF2-related genes, providing new avenues for research and therapeutic intervention.

Several bioactive compounds have been tested in patients with T2DM for their ability to modulate the NRF2-KEAP1 pathway, thereby inducing key antioxidant enzymes to suppress disease-related inflammation and oxidative stress.

Twenty-four patients with obesity and T2DM undergoing insulin therapy were randomly assigned to receive either exenatide (a glucagon-like peptide-1 receptor agonist) or a placebo twice daily for 12 weeks. The study demonstrated that exenatide treatment significantly increased expression of *NQO1* and *HMOX1*, as well as the protein levels of NQO1 in PBMCs (p < 0.05) [39].

Another study aimed to explore the mechanisms underlying the potential of curcumin in diabetic kidney disease. Curcumin, a polyphenol compound found in turmeric (*Curcuma longa*), and known as an NRF2 activator in various cells and animals [40], was administered to fourteen T2DM patients, seven of whom had diabetic nephropathy with albuminuria as a hallmark. The intervention significantly reduced urinary albumin excretion without affecting the metabolic control, namely glycaemia, and lowered concentrations of c-LDL in plasma, while simultaneously enhancing the NRF2 pathway, specifically upregulating proteins such as NQO1 (p < 0.05) in lymphocytes.

In another randomized, double-blind, placebo-controlled crossover study, a combination of *trans*-resveratrol and hesperetin (tRES-HESP) was evaluated for its potential to reverse insulin resistance, improve dysglycemia, and reduce low-grade inflammation in overweight and obese individuals. The study included 20 participants who were highly overweight or obese (body mass index (BMI) ≥ 27.5 kg/m^2^) and 11 participants who were obese (BMI ≥30 kg/m^2^). Treatment with tRES-HESP reduced several markers of damage associated with T2DM, including plasma methylglyoxal levels and the expression levels of several genes. The study identified a negative correlation between plasma methylglyoxal levels and *NQO1* mRNA levels in PBMCs (*r* = −0.68, p < 0.01). It also decreased fasting plasma glucose, which also negatively correlated with *NQO1* expression (*r* = −0.50, p < 0.05) [41].

Bromocriptine-quick release (bromocriptine-QR), a sympatholytic dopamine D2 agonist, was evaluated in T2DM patients whose glycemia levels were not optimally controlled with glucagon-like peptide 1 receptor agonists [42]. Fifteen patients with T2DM and cardiovascular disease were enrolled in a 4-month observational study of circadian-timed bromocriptine-QR administered within 2 h of waking in the morning, including assessment of a wide range of immune, pro-oxidative, and pro-inflammatory biochemical pathways and genes’ expression associated with cardiovascular disease progression in T2DM. After 4 months of treatment, significant reductions in mRNA levels of key oxidative stress response genes were observed in PBMCs. Notably, ∼40 % decreases were detected in *NQO1* (p < 0.05) and *HMOX1* (p < 0.001) expression levels. This reduction in the expression of oxidative stress response genes could be indicative of a reduction of oxidative stress in T2DM by the drug [42].

These findings indicate the potential of exenatide, curcumin, tRES-HESP and bromocriptine-QR in mitigating oxidative and inflammatory stress pathways in T2DM patients and individuals with obesity, via the modulation of NRF2-related genes. Expression levels of the NRF2-regulated genes could potentially serve as a read-out for response to treatment with these drugs and supplements, most likely in combination with other clinical biomarkers.

Two other studies have reported elevated levels of oxidative stress markers in T2DM [43,44]. Notably, T2DM and inflammation of the gums, periodontitis (PDT), were associated with oxidative stress. A comparative study examined patients with comorbid T2DM-PDT, individuals with PDT alone, T2DM alone, and healthy controls. PBMCs from T2DM and T2DM-PDT patients exhibited increased GCL activity, observed exclusively in T2DM-PDT patients, and elevated GCL-catalytic subunit protein levels [43]. Despite these changes, mRNA levels of *GCLC* and *HMOX1*, remained consistent, as did those in healthy controls [43]. However, when PBMCs from T2DM-PDT patients were compared to those from PDT patients, levels of NRF2-target genes were increased in T2DM-PDT patients (GCLC (p < 0.01), GCLM and HMOX1 (p < 0.05)).

The assumed hereditary, but mostly lifestyle-related nature of T2DM, has been found to be associated with an increased risk of developing this condition. Individuals with a first-degree relative with T2DM (FDRT) serve as a natural model to investigate susceptibility factors contributing to T2DM, many of which remain poorly understood. To address this, a study was conducted to examine the role of oxidative stress and inflammatory responses as mediators of the heightened risk for T2DM, enrolling nine normoglycemic men matched one-to-one with, or nine without, a family history of T2DM. The study participants with no former history of smoking, alcohol consumption, overt disease, medication intake, atypical dietary habits, hospitalization or surgery (6 months) and weight change >5 % (3 months), were matched for BMI and insulin sensitivity [44]. When measured in PBMCs, levels of the NRF2 target gene *TXNRD1* (p between group <0.01, p time × group <0.01), were upregulated in FDRT-derived PBMCs after meal ingestion [45].

In conclusion, oxidative stress and disrupted redox homeostasis play a crucial role in T2DM and its comorbid conditions. Monitoring oxidative stress responses in patients with comorbid conditions may help optimize treatment strategies and improve overall disease management. Additionally, individuals with a family history of T2DM may exhibit altered oxidative and inflammatory responses. Identifying these markers could aid in early risk assessment and stratification of individuals predisposed to T2DM, particularly those with a family history. However, sufficiently powered prospective cohort studies are needed to confirm the limited existing evidence.

Metabolic syndrome (MS) is a condition with a cluster of risk factors linked to (abdominal) obesity that encompasses insulin resistance, hypertension, and hyperlipidemia, with chronic inflammation and oxidative stress playing key roles in its development and its associated risks to T2DM and cardiovascular diseases [46]. The mRNA expression level of *HMOX1* in PBMCs was significantly elevated in 30 subjects with MS as compared to 14 controls, showing an increase of 185 % [47]. In another study, subjects with MS received different dietary interventions for over 12 weeks, including a high-saturated fatty acid (HSFA) diet. The expression of *TXNRD1* in PBMCs significantly increased after the HSFA meal (p < 0.05), whereas no changes were observed following other dietary interventions [48].

### 3.5 Cardiovascular diseases

Cardiovascular diseases (CVDs) remain the leading global cause of mortality, responsible for approximately 17.9 million deaths annually, with ischaemic heart disease and strokes accounting for over 80 % of these fatalities [49]. These disorders of the heart and blood vessels, including hypertension and heart failure, are exacerbated by oxidative stress. Elevated reactive oxygen species (ROS) levels, derived from mitochondrial dysfunction, impaired endothelial function, and environmental factors like pollution, drive intracellular calcium overload, inflammation, and vascular damage, accelerating CVD progression [50]. Recent epidemiological data highlight that one-third of CVD deaths occur prematurely in individuals under 70 years of age, underscoring the urgency of addressing modifiable risk factors like oxidative stress [49]. Therapeutic strategies targeting antioxidant pathways, such as NRF2 activation, offer emerging approaches to mitigate redox imbalance and improve outcomes.

Some studies aimed to investigate the role of the NRF2-induced genes/proteins in specific cardiovascular conditions and/or following traditional pharmacological approaches. For example, in carotid endarterectomy samples, specifically from atherosclerotic plaques, gene expression levels were compared to those from the surrounding healthy tissues of the same patients' arterial specimens. The analysis revealed a nearly 2-fold increase in *TXNRD1* mRNA expression in the atherosclerotic plaques relative to the adjacent healthy arterial tissue (p < 0.05) [51].

In abdominal aortic aneurysm (AAA) patients, tissue samples were obtained from individuals undergoing surgical AAA repair. These patients were divided into two groups, i.e., those treated with simvastatin, a cholesterol-reducing drug belonging to the group of statins, and those not treated with statins during a period of 6 months preceding surgery, with the groups being matched by AAA diameter and age. While HMOX1 protein levels were significantly higher in the simvastatin-treated group (p < 0.05), the expression of the NRF2 target genes *HMOX1, NQO1,* and *GCLM* in AAA tissue did not show significant differences between the treated and untreated groups [52].

Patients undergoing cardiopulmonary bypass (CPB) surgery, implicating exposure to non-endothelial surfaces, had blood samples collected preoperatively, during CPB, and 24 h postoperatively, showed a significant 2-fold increase in *HMOX1* expression upon initiation of CPB (p < 0.01), while *HMOX1* expression decreased by 40 %, returning approximately to preoperative levels 24 h after surgery [53].

On the other hand, in coronary artery disease (CAD), the mRNA levels of the antioxidant enzyme *NQO1* in PBMCs showed no significant difference as compared to control samples [54], highlighting either a systemic adaptation to the disease or that distinct pathways are activated in a disease/pathology-specific mode.

### 3.6 Chronic kidney diseases

Kidney Disease Improving Global Outcomes (KDIGO) organization defines chronic kidney disease (CDK) as abnormalities of kidney structure or function, lasting at least 3 months and carrying health implications. Untreated CDK leads to progressive kidney failure [55], and diminished kidney functions increasing the risk of cardiovascular-related mortality [56]. Oxidative stress seems to play a key role in disease progression with oxidative stress markers rising from the early stages of CKD as renal function deteriorates [57]. Clinical data on NRF2 abundance and activity in patients who suffer from CKD remain limited, with most analyses based on kidney biopsy samples. Under normal conditions, NRF2 is predominantly expressed in renal tubules but in CKD patients, it also has been found in the glomeruli [[58], [59], [60]].

Our search identified five articles, each examining *NQO1* and/or *HMOX1* gene expression or their protein products in patients who suffered from CKD. Two of these studies compared patients with CKD to healthy volunteers [61]. Shen et al. demonstrated a statistically significant increase in *NQO1* expression (p < 0.01 for *NQO1/*Actin beta (*ACTB*); p < 0.001 for *NQO1/*60S ribosomal protein L41 (*RPL41*) and p < 0.001 for *NQO1/*TATA-box binding protein (*TBP*)) in monocytes isolated from patients with non-haemodialysis-dependent CKD. In patients undergoing haemodialysis, this effect was less pronounced, with elevated *NQO1* mRNA levels observed only when normalised to TBP (p < 0.05 for *NQO1/TBP*) [61]. Pedruzzi et al. documented a decrease in *NQO1* expression in PBMCs of CKD patients compared to healthy individuals (p < 0.01)*.* Additionally, their study examined the *HMOX1* gene, which showed comparable mRNA levels between CKD patients and healthy controls [62].

The remaining three studies [[63], [64], [65]] focused exclusively on patients with CKD. The first study, a placebo-controlled RCT, evaluated the effectiveness of Brazil nut supplementation in activating the NRF2 pathway in haemodialysis patients. The results showed a significant increase in *NQO1* expression in PBMCs after supplementation in the treated group (p < 0.001), while in the placebo group no significant changes were observed [63]. The second study, another placebo-controlled RCT including a crossover in haemodialysis patients, investigated the effects of resistant starch (RS) enriched cookies on mRNA and protein levels. The findings suggested that RS-enriched cookies could be a beneficial nutritional strategy to reduce indoxyl sulphate levels derived from gut microbiota and attenuate inflammation in haemodialysis patients. The study reported that the period effect and the carryover effect were significant for the increased NQO1 protein levels in PBMCs (p < 0.05). However, such a change was not observed in *NQO1* gene expression. Additionally, baseline analysis revealed a positive association between *NFEL2L* and *NQO1* expression (p < 0.01). Importantly, *HMOX1* expression remained unchanged [64]. The third study, using SFN, was conducted in CKD patients not requiring renal replacement therapy and demonstrated an elevated *NQO1* mRNA level in monocytes after SFN treatment (p < 0.05) compared to baseline (which was lower than in the placebo group). No effect in the placebo group, and no significant difference between the placebo and the treatment groups were observed. Furthermore, the same group exhibited only a trend (p > 0.05) towards increased *HMOX1* expression after the intervention [65].

### 3.7 Hereditary blood disorders

Sickle cell disease (SCD) is one of the most widespread and serious inherited diseases worldwide. Between 2000 and 2021, the number of people with SCD increased to 7.74 million globally, with most deaths occurring in African countries and a major cause of mortality in the 5- to 14-year age group [66,67].

A key contributor to the pathophysiology of SCD is elevated plasma haem levels resulting from chronic red blood cell haemolysis, which leads to a marked increase in HMOX1 levels. NRF2 also regulates iron mobilization during haemoglobin degradation, further underscoring its importance. Finally, the ability of NRF2 to regulate glutamine metabolism highlights its potential as a target for developing small-molecule activators to treat SCD.

Our search identified a single article reporting a phase I clinical trial involving treatment in patients homozygous for sickle haemoglobin. Patients received three different doses (50 g, 100 g, and 150 g) of broccoli sprout homogenate (BSH), containing the NRF2 activator SFN. The effects were assessed by comparing measurements taken at baseline (day 0), the last day of ingestion (end of treatment), and following a washout period. For participants receiving the 150 g daily dose of BSH orally for 21 days (but not the lower doses), *HMOX1* expression was significantly upregulated in whole blood from pre-to post-treatment (p < 0.05). In contrast, the measurement of *NQO1* mRNA levels did not show significant differences among treatments or doses [68].

Our search also retrieved a study summarizing the involvement of the NRF2 pathway in β-thalassemia/Haemoglobin E (HbE) patients [69]. Patients with β-thalassemia/HbE haemoglobinopathy exhibit a broad range of clinical symptoms, often leading to severe transfusion-dependent thalassemia major. Although routine blood transfusions and iron chelation therapy have improved life expectancy, complications related to transfusional iron overload remain common [70]. In this study, β-thalassemia patients received regular blood transfusions and underwent iron chelation therapy with desferrioxamine [69]. Gene expression analysis of PBMCs in patients revealed an upregulation of *GCLC* (p < 0.05), while *HMOX1* levels remained unaffected; protein levels corresponded to the expression of both genes (GCLC: p < 0.01).

### 3.8 Cancer

Among NCDs, cancer causes 23 % of deaths worldwide. One in five people will develop cancer during their lifetime, whereas the mortality rates in men and women are 1 in 9 and 1 in 12, respectively. The latest comprehensive global cancer statistics were published by the International Agency for Research on Cancer (IARC), concerning data collected in 2022 and available in February 2024 [71], reporting nearly 20 million new cancer cases and 9.7 million deaths from cancer. Demographics-based predictions due to the ageing of the population estimate up to 35 million new annual cancer cases by 2050 [71].

Systems medicine, which applies a mechanism-based approach to disease for identifying therapeutic targets beyond a hypothesis-driven approach, has evidenced the altered NRF2 interactome in advanced cancers and neoplasms [11].

Reszka et al. used whole blood samples to investigate the NRF2 interactome, studying 122 urinary bladder cancer patients and comparing the obtained data to 115 healthy individuals [72]. The *GCLM* expression was significantly upregulated (p < 0.05) in blood samples collected from cancer patients [72]. Multivariable analysis of gene expression, adjusted by age, BMI, and smoking/drinking habits, revealed statistically significant increases (p < 0.01) in the *HMOX1* and *NQO1* expression levels. The association between gene expression and DNA damage, with the same adjustments applied, demonstrated significant increases in the *GCLC* (p < 0.01) and *GCLM* (p < 0.001) expression levels [72].

In another study, 14 patients with advanced solid tumours and lymphomas were enrolled in a first-in-human phase I clinical trial using bardoxolone methyl, a semi-synthetic NRF2 inducer, and were screened for *NQO1* transcript levels in PBMCs isolated from blood samples collected before and post-treatment [73]. As measured on day 2 and day 22 of the first cycle of the treatment, significant increases (p < 0.001) over the baseline *NQO1* expression levels were detected [73].

HMOX1 was assessed in cluster of differentiation (CD)45^+^CD3^−^CD19^−^CD56^−^ monocytes isolated from melanoma patients, stratified by differential expression of CD14 and CD16 in these blood cells [74]. Fluorescence-activated cell sorting of the permeabilised cells stained with fluorescein-conjugated antibody anti-HMOX1 revealed the presence of three monocyte subsets and the confirmation sets of 47 and 45 patients, respectively. The highest HMOX1 levels characterized the CD14^+^CD16^+^ monocytes, significantly increased as compared to the CD14^+^CD16^−^ monocytes (p < 0.001) and the CD14^Dim^CD16^+^ monocytes (p < 0.05). Importantly, a Kaplan–Meier analysis revealed lower survival in melanoma patients characterized by high HMOX1 levels (different cut-off values), regardless of the monocyte subtype [74]. Ma et al. isolated PBMCs from 35 advanced-stage chronic myeloid leukaemia (CML) patients treated with the tyrosine kinase inhibitor imatinib [60]. Individuals insensitive to imatinib had significantly higher (p < 0.05) *HMOX1* expression levels than the responders [60]. Miyazaki et al. reported lower HMOX1 protein levels in blood cancer cells isolated from 12 acute myeloid leukaemia patients, compared to the levels detected in monocytes isolated from four healthy donors; in both cell types, the measured HMOX1 levels were normalised to the levels assessed in the reference U937 lymphoma cells [75].

By comparing datasets on mRNA levels supported by clinically relevant information from three independent Gene Expression Omnibus (GEO) patient cohorts, Wang et al. [76] attempted to predict risk-scoring genetic signatures relevant to overall survival in 604 diffuse large B-cell lymphoma (DLBCL) patients (in the original studies on B-cell derived tumour samples, gene expression profiling was performed with the use of microarrays [77] or next-generation sequencing (NGS) [78]). The univariate Cox regression analysis revealed that the upregulated *GCLC* expression significantly reduced (p < 0.001) patient survival [76,77,79,80].

As evidenced by (relatively scarce) studies on blood samples obtained from cancer patients or samples of blood cell-derived solid tumours (leukaemia or lymphomas), alterations in the NRF2 interactome and/or expression may have not only prognostic relevance but also pose attractive therapeutic targets for new drugs/drug combinations. Retrospective analyses of clinical datasets, careful examination of the available biobank samples, and future state-of-the-art clinical studies are undoubtedly needed to improve the understanding of the roles that genes transcriptionally regulated by NRF2 play in carcinogenesis and cancer resistance to therapy.

### 3.9 Other diseases

Studies on diseases that do not fall into the previous categories have also been analysed in relation to the possible implication of NRF2-related biomarkers. These include cholestatic liver injury, and autism spectrum disorder (ASD). While these conditions differ significantly in their pathophysiology and clinical manifestations, they share several features and underlying mechanisms, particularly related to immune dysregulation, oxidative stress, and inflammation [46,[81], [82], [83]].

#### 3.9.1 Cholestatic liver disease

This disease is characterized by impaired bile flow caused by the obstruction of the duct or dysfunction in bile production, leading to the accumulation of toxic bile acids within the liver, which triggers inflammation, oxidative stress, and hepatocellular damage [81]. Blood samples were collected from patients and healthy subjects and the expression of *HMOX1* was found to be significantly elevated in patients with cholestatic liver injury (p < 0.001). These findings, along with evidence from animal models of cholestatic liver injury and cell culture experiments, revealed a previously unrecognized role of NRF2 signalling in exacerbating liver injury in cholestatic disease [84].

#### 3.9.2 Autism spectrum disorder

ASD is characterized by persistent deficits in social communication and interaction, restricted-repetitive behaviours, and is often associated with immune system dysfunction and strong inflammatory states [83]. SFN was administered orally as a dietary supplement to six children with ASD for two weeks. The expression of the NRF2-target genes *NQO1* and *HMOX1* was elevated in PBMCs isolated from patients with ASD compared to their baseline levels, but it did not reach statistical significance [85].

## 4 Discussion

Non-communicable diseases, including cardiovascular diseases, type 2 diabetes, neurodegenerative disorders, and cancer, are the leading causes of morbidity and mortality worldwide. The initiation and progression of these heterogeneous diseases are associated with (among others) redox imbalance, which often leads to persistent oxidative stress and chronic inflammation, as well as a dysregulation of the NRF2 pathway. NRF2 plays a critical role in maintaining cellular redox homeostasis and promoting anti-inflammatory responses, and thus lends itself as a promising therapeutic target for the treatment of various NCDs.

In this review, we have gathered evidence on the systemic read-out of NRF2 activity based on the previously defined robust panel of six genes/proteins in various NCDs [9]. Biomarkers play a crucial role in diagnosing disease, informing treatment decisions or stratifying patients, and thus are essential instruments in clinical routine for disease prevention and prognosis or treatment outcome.

**Fig. 5:**
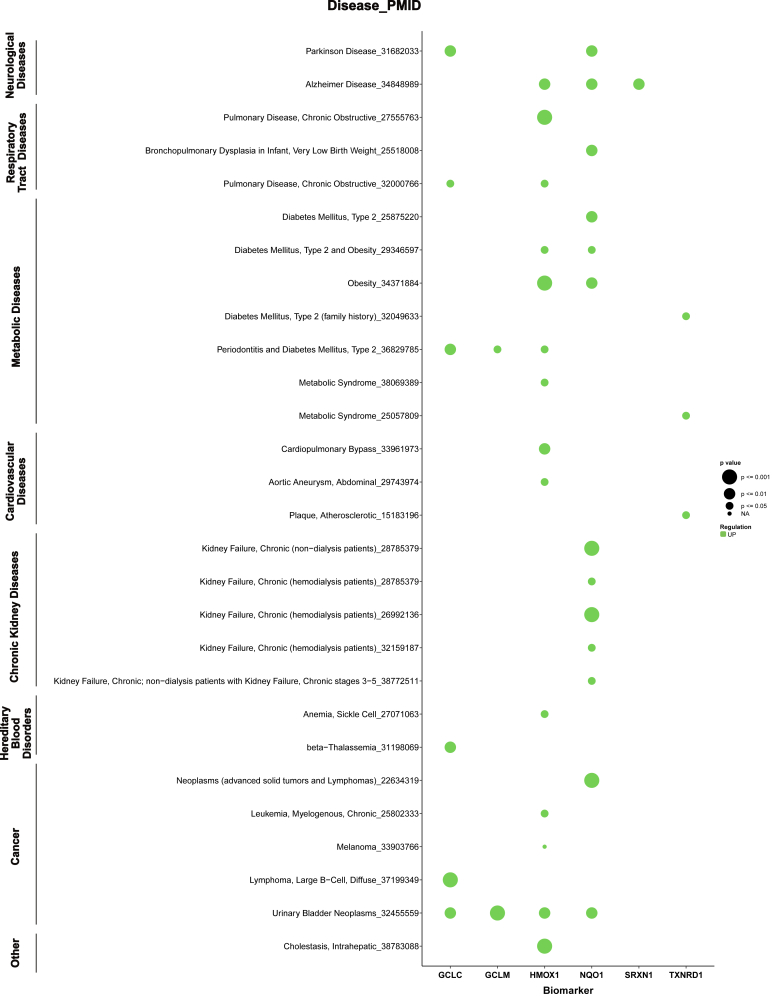
**NRF2 Pathway activity in non-communicable diseases.** Bubble chart showing upregulated expression of six key NRF2-related biomarkers (GCLC, GCLM, HMOX1, NQO1, SRXN1, TXNRD1) [5]. Green colour indicates upregulation. Bubble size is proportional to the statistical significance (strength of the p-value). Blank cells indicate that the biomarker was not upregulated, or was not measured, in that disease (see also Fig. 6 and Table S1).

Data characterising the systemic phenotype of NRF2 signalling comes from the Human Protein Atlas, which has information on the expression of *GCLC*, *GCLM*, *HMOX1*, *NQO1*, *SRXN1* and *TXNRD1* in different blood cells (Fig. 4A). While *HMOX1* is highly expressed in monocytes, the expression of *TXNRD1* is high across all measured cell types and peaks in granulocytes. In NK-cells, the genes regulating glutathione metabolism, i.e., *GCLC* and *GCLM* as well as *NQO1* and *TXNRD1,* are highly expressed. Although expression levels of the hallmark gene panel vary across blood cell types, all genes within the panel are expressed (Fig. 4A). Conversely, protein expression levels in PBMCs are highest for HMOX1, followed by TXNRD1 and NQO1 (Fig. 4B), supporting PBMCs as a reliable source for monitoring NRF2 signalling. Indeed, our literature search identified significant increases in HMOX1 (p < 0.001) in PBMCs isolated from COPD patients [34] and obese subjects [41], measured at the gene expression and protein levels (Table S1, Fig. 5). The levels of the *NQO1* gene were significantly upregulated (p < 0.001) in PBMCs of patients diagnosed with advanced solid tumours and lymphomas [73], as well as haemodialysis patients suffering from CKD [63], as analysed by RT-qPCR (Table S1, Fig. 5).

The pattern of HMOX1 and NQO1 expressed at high levels in NCDs is reflected in our analysis, where these NRF2-regulated enzymes emerged as the most frequently studied biomarkers across all diseases investigated, showing significant upregulation in most NCDs examined (Fig. 5). Our search identified NQO1 as elevated (p < 0.01) in patients with neurological diseases, including Parkinson disease and Alzheimer disease. This is in agreement with previous reports highlighting alterations of NQO1 in the brain and linking those changes to the pathophysiology of neurological disorders [86].

**Fig. 6:**
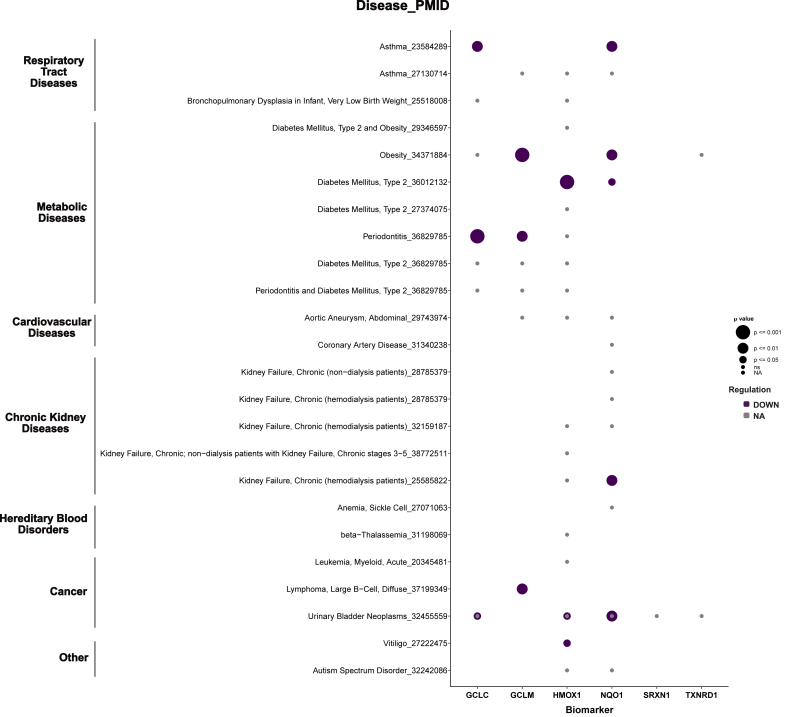
**NRF2 Pathway activity in non-communicable diseases.** Bubble chart showing the expression of six key NRF2-related biomarkers (GCLC, GCLM, HMOX1, NQO1, SRXN1, TXNRD1) [5]. Purple denotes downregulation, and grey indicates an unknown direction of regulation. Bubble size is proportional to the statistical significance (strength of the p-value). Blank cells indicate that the biomarker was not downregulated/lacked statistical significance, or was not measured in that disease (see also Fig. 5 and Table S1).

Within respiratory tract diseases, HMOX1 was most often found to be upregulated, albeit the strength of evidence within COPD patients differed (Fig. 5). In contrast, we observed that NRF2 target genes were downregulated in asthma (Fig. 6). This may reflect asthma's typical onset in childhood, suggesting that the protective regulatory effects of NRF2 could have diminished by adulthood in the studied population.

The heart is particularly vulnerable to oxidative stress due to its high reactive species production from mitochondrial oxidative phosphorylation, β-oxidation of fatty acids, and other cellular pathways, coupled with relatively low endogenous antioxidant levels. This susceptibility is further exacerbated by conditions like ischemia and reperfusion, making cardiac tissue prone to oxidative damage [87]. NRF2 plays a crucial role in cellular defence, yet its role in cardiac health remains underexplored in comparison to other contexts [88]. Despite its potential significance, studies on NRF2 target genes in CVDs are scarce as we confirmed herein. *HMOX1* is the most frequently reported gene with significant changes, but only two studies highlight its involvement [52,53]. Aside from one study noting increased *TXNRD1* expression [51], other NRF2-related markers remain unchanged. Most research focuses on disease susceptibility rather than the potential of NRF2-related genes as indicators of cardioprotective treatment effects (e.g., simvastatin) [52]. Furthermore, the diversity of biological samples beyond PBMCs may introduce confusion and bias in overall assessments and lead to more significant changes in systemic NRF2-related genes.

On the other hand, NRF2-related genes have been extensively studied in the context of T2DM, yielding more robust and consistent associations compared to CVDs, where findings remain relatively limited despite the presence of related pathophysiological mechanisms.

In T2DM, studies have shown that NRF2-regulated genes such as NQO1, HMOX1, GCLC, and TXNRD1 serve as important biomarkers for therapeutic response, oxidative stress modulation, and disease progression. Notably, NQO1 and HMOX1 have been identified as predictors of therapeutic response to pharmacological treatments, although their expression levels can vary - increased or decreased - depending on the presence of comorbidities (yet they remain strongly implicated). Moreover, TXNRD1 has emerged as a promising prognostic marker for T2DM risk, particularly in familial forms of the disease [45], while *GCLC* (p < 0.01) and *GCLM* as well as *HMOX1* (both p < 0.05) appear to play a role under specific clinical conditions of periodontitis accompanying T2DM [43].

HMOX1 has also been reported as upregulated in two hereditary blood diseases, sickle cell disease and beta-Thalassemia, where NRF2 activation can potentially alleviate disease severity by reducing oxidative damage, inhibiting haemoglobin S polymerization, and promoting foetal haemoglobin expression [89].

A urinary bladder cancer study found *GCLC, GCLM, HMOX1*, and *NQO1* to be upregulated. This was the most comprehensive study identified, as it was the only one to measure all six biomarkers. [72]. Aberrant expression of NRF2 within cancer might lead to an increased cell proliferation due to a disturbed redox homeostasis, posing a survival benefit to cancer cells, and thereby leading to a decreased survival rate of cancer patients [90]. Unlike other NCDs, where enhancing NRF2 signalling is therapeutically attractive, in cancer, the pathway's overactivation must be suppressed to provide a viable treatment strategy. From the diagnostic point of view, identification of the easily accessible subset CD14^+^CD16^+^ of monocytes offers a valuable tool for melanoma patient stratification in HMOX1 levels to predict patient survival [74].

Similarly, in kidney diseases, a prolonged upregulation of the NRF2 signalling pathway was shown to have detrimental effects [91]. These and further studies hint at the critical – and so far, unanswered – questions of the right timing and spatial context of NRF2 pathway modulation for translational applications.

In addition to well-established disease groups, NRF2 signalling has also been implicated in several heterogeneous conditions such as metabolic syndrome, cholestatic liver injury, vitiligo, and autism spectrum disorder [[76], [77], [78], [79], [80]]. Despite their clinical diversity, these conditions share core pathological features including chronic inflammation, oxidative stress, and immune dysfunction, all of which being considered hallmarks of disrupted redox homeostasis [[72], [73], [74], [75]]. The consistent alteration of NRF2-related gene expression across studies and pathophysiologies suggests a broader systemic role for NRF2 beyond classical metabolic or degenerative disorders. However, while preliminary findings are promising, the current body of evidence is limited by small sample sizes and a lack of mechanistic validation. Future studies are needed to assess the diagnostic, prognostic, and therapeutic utility of NRF2-associated pathways across these diverse disease states.

**Fig. 7:**
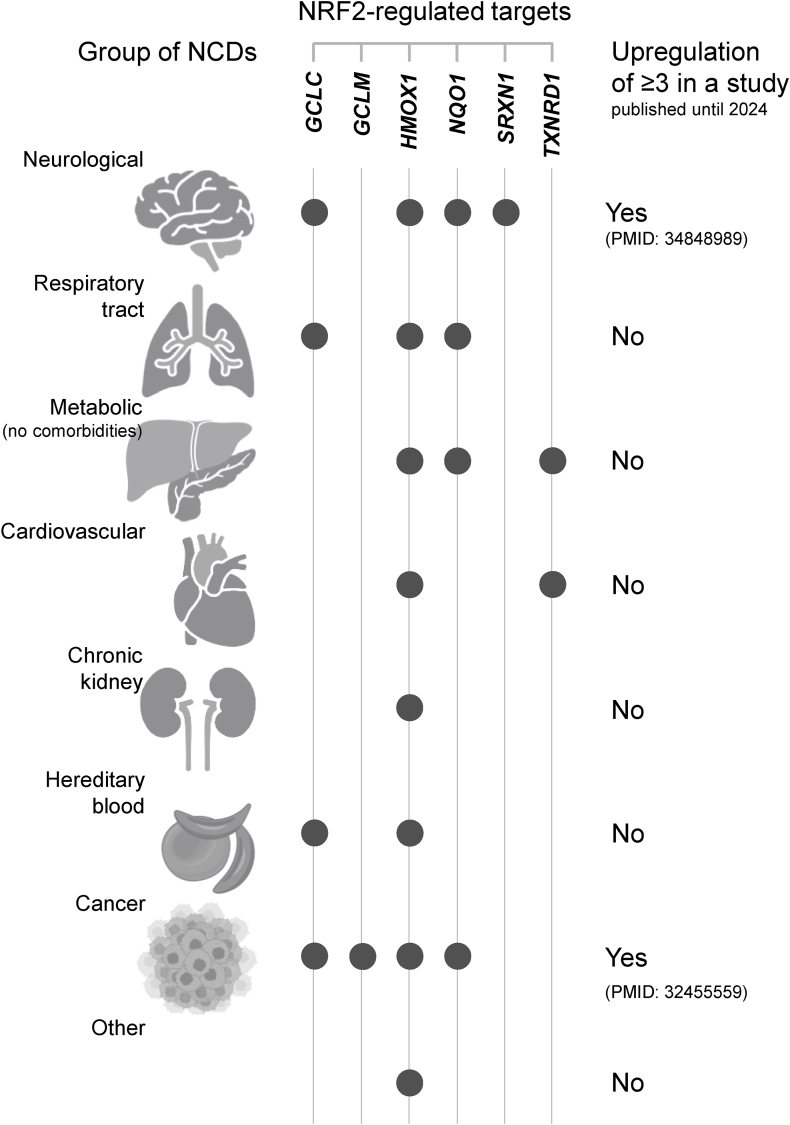
**Graphical summary of the main results.** In given groups of non-communicable diseases (NCDs), upregulation in gene/protein targets of NRF2 is shown using bubbles. Until 2024, two human studies, PMID: 34848989 in neurological diseases [24] and PMID: 32455559 in cancer [72], reported the concomitant upregulation in panels of three biomarkers, in line with the reporting policy proposed in our 2024 literature review [9]; see also Fig. 5 and Table S1.

The limitations of this study include its exclusive reliance on published studies from two major databases, excluding data from sources such as clinical trial registries. However, our objective was to prioritise peer-reviewed, evidence-based research outcomes (Table S1). Additionally, the restrictive search criteria may have excluded potentially relevant studies. While this review includes a limited number of 36 studies, the findings are drawn from a diverse range of non-communicable diseases across multiple organ systems (Fig. 7). This breadth suggests that the fundamental challenge is not the number of studies, but the inherent biological complexity of establishing a unified NRF2 signature across distinct pathologies. Moreover, we did not perform a comprehensive statistical meta-analysis that could lead to somewhat missed assumptions.

We provide evidence supporting the six NRF2 biomarkers proposed in 2024, originally derived from *in vitro* and *in vivo* research [9], can serve as a systemic read-out of NRF2 signalling across a variety of NCDs, but their utility in clinical prediction remains largely unknown. While there is no consistent expression pattern across the investigated NCDs with both up- and downregulated NRF2 targets, it becomes evident that the spatio-temporal context of a specific NCD warrants further investigation. The relationship between transcript concentration and protein abundance is complex, governed by post-transcriptional regulation including translation rates, protein degradation, and subcellular localisation [92]. Integrating high-throughput transcriptomic and proteomic data is therefore crucial to elucidate the full context of the NRF2-mediated responses.

Further factors that are contributing to this uncertainty are sex, age, lifestyle and nutritional aspects. Numerous studies have shown that upon aging, the production of oxidants is increased while the activity of antioxidant enzymes vanishes with a simultaneous decline in adaptive response to redox imbalance via NRF2 [93]. Furthermore, the impact of nutrition on health has gained considerable attention, with phytochemicals implicated in reducing disease risk. The most prominent example of this NRF2-mediated nutraceutical effect is sulforaphane, an electrophile that has shown to activate NRF2 through direct interaction with the reactive cysteine of KEAP1 [94]. Thus, as discussed previously [9], inter-individual variability in NRF2 signalling adds to the dynamics and provides a challenge in establishing a universal level of NRF2 activity indicative of disease.

## CRediT authorship contribution statement

**Monika Jakubowska:** Writing – review & editing, Writing – original draft, Visualization, Methodology, Investigation, Data curation, Conceptualization. **Vera Marisa Costa:** Writing – review & editing, Writing – original draft, Investigation, Data curation, Conceptualization. **Wojciech Krzeptowski:** Writing – review & editing, Writing – original draft, Methodology, Investigation, Data curation, Conceptualization. **Pia Pužar Dominkuš:** Writing – review & editing, Writing – original draft, Methodology, Investigation, Data curation, Conceptualization. **Marlene Santos:** Writing – review & editing, Writing – original draft, Investigation, Data curation, Conceptualization. **Birsen Can Demirdöğen:** Writing – review & editing, Writing – original draft, Investigation, Data curation, Conceptualization. **Şermin Genç:** Writing – review & editing, Writing – original draft, Investigation, Data curation, Conceptualization. **Ioannis P. Trougakos:** Writing – review & editing, Writing – original draft, Investigation, Conceptualization. **Katja M. Kanninen:** Writing – review & editing, Writing – original draft, Investigation, Conceptualization. **Brigitte M. Winklhofer-Roob:** Writing – review & editing, Writing – original draft, Investigation. **Ian M. Copple:** Writing – review & editing, Writing – original draft. **Antonio Cuadrado:** Writing – review & editing, Writing – original draft. **Vita Dolžan:** Writing – review & editing. **Christina Morgenstern:** Writing – review & editing, Writing – original draft, Project administration, Methodology, Investigation, Data curation, Conceptualization.

## Declaration of competing interest

None of the authors have competing interest to declare.

## Data Availability

No data was used for the research described in the article.
